# ORMDL mislocalization by impaired autophagy in Niemann-Pick type C disease leads to increased *de novo* sphingolipid biosynthesis

**DOI:** 10.1016/j.jlr.2024.100556

**Published:** 2024-05-06

**Authors:** Ryan D.R. Brown, Usha Mahawar, Binks W. Wattenberg, Sarah Spiegel

**Affiliations:** Department of Biochemistry and Molecular Biology, Virginia Commonwealth University School of Medicine, Richmond, VA, USA

**Keywords:** ORMDL, sphingolipid biosynthesis, NPC1

## Abstract

Niemann-Pick type C1 (NPC1) disease is a rare neurodegenerative cholesterol and sphingolipid storage disorder primarily due to mutations in the cholesterol-trafficking protein NPC1. In addition to catabolic-derived sphingolipids, NPC1 dysfunction also leads to an increase in de novo sphingolipid biosynthesis, yet little is known about the cellular mechanism involved. Although deletion of NPC1 or inhibition of the NPC1 sterol binding domain enhanced de novo sphingolipid biosynthesis, surprisingly levels of the ORMDLs, the regulatory subunits of serine palmitoyltransferase (SPT), the rate-limiting step in sphingolipid biosynthesis, were also greatly increased. Nevertheless, less ORMDL was bound in the SPT-ORMDL complex despite elevated ceramide levels. Instead, ORMDL colocalized with p62, the selective autophagy receptor, and accumulated in stalled autophagosomes due to defective autophagy in NPC1 disease cells. Restoration of autophagic flux with N-acetyl-L-leucine in NPC1 deleted cells decreased ORMDL accumulation in autophagosomes and reduced de novo sphingolipid biosynthesis and their accumulation. This study revealed a previously unknown link between de novo sphingolipid biosynthesis, ORMDL, and autophagic defects present in NCP1 disease. In addition, we provide further evidence and mechanistic insight for the beneficial role of N-acetyl-L-leucine treatment for NPC1 disease which is presently awaiting approval from the Food and Drug Administration and the European Medicines Agency.

Niemann-Pick disease type C1 is a rare autosomal-recessive lipid storage disease resulting from loss-of-function mutations mainly in *NPC1* or *NPC2* to a lesser extent. The progressive hepatosplenomegaly, neurodegeneration, and morbidity in these patients (1) have been attributed to the excessive accumulation of unesterified cholesterol, sphingosine, and other sphingolipids, predominantly in the brain, and in other tissues such as the liver and spleen (2, 3, 4, 5, 6, 7, 8). It has been elegantly demonstrated that NPC proteins act sequentially in late endosomes/lysosomes (LE/LY), where the soluble NPC2 protein binds unesterified cholesterol and transfers it to the luminal sterol-binding site of membrane-associated NPC1, which then facilitates its transport out of the LE/LY for esterification in the ER (9). Accumulation of cholesterol causes a secondary reduction in activity of acidic sphingomyelinase (10) and/or other lysosomal enzymes responsible for degrading complex sphingolipids to sphingosine. More recently, it has been suggested that NPC1 is also involved in sphingosine export from LE/LY compartment (11), providing an additional explanation for the backup of lysosomal sphingolipids. In addition to catabolic-derived sphingolipids, it has long been known that patients with NPC1 and NPC1 mutant mice also have a dramatic accumulation of dihydrosphingosine (5, 7), a sphingolipid metabolite exclusively generated in the de novo biosynthetic pathway. However, although the causative relationship between NPC mutations and complex sphingolipid accumulation in LE/LY has been extensively studied, little is known about the mechanism behind the increased *de novo*-derived dihydrosphingosine.

De novo biosynthesis of sphingolipids begins in the ER with serine palmitoyltransferase (SPT) catalyzing the condensation of serine with palmitoyl-CoA to generate 3-ketodihydrosphingosine (12). SPT is a complex consisting of two large subunits SPTLC1 and SPTLC2 (or SPTLC3) and one of the two small subunits SPTSSA or SPTSSB, which enhance enzyme activity and determine acyl-CoA substrate usage (13). The SPT rate-limiting step is homeostatically controlled by the negative regulator ORMDL proteins (ORMDL1, -2, and -3), forming an intrinsic part of the SPT complex (ORMDL-SPT) (14, 15, 16). High levels of intracellular ceramide are sensed by ORMDL causing a conformational change in the N-terminus of ORMDL leading to a blockade of the acyl-CoA binding pocket and inhibiting SPT activity and de novo biosynthesis (16). Once formed, 3-ketodihydrosphingosine is irreversibly and rapidly reduced to dihydrosphingosine, which is N-acylated by ceramide synthase (CerS 1–6 with affinities for various fatty acyl-CoA) to form dihydroceramides that are desaturated at the C4 position to yield ceramides. Dihydroceramide and ceramide can either be transported out of the ER to the trans-Golgi by ceramide transfer protein (CERT) for conversion to sphingomyelin or via vesicular transport to the cis-Golgi to form glucosylceramide and subsequently complex glycosphingolipids.

As the cellular mechanism responsible for the increase in de novo sphingolipid biosynthesis due to NPC1 mutation or deletion is still unknown, in this work, we set out to examine the involvement of the sphingolipid sensor ORMDL, the known regulator of SPT activity (14, 15, 16, 17). Surprisingly, we found that although the ORMDL level is increased in NPC1-deleted cells, it is decreased in the ORMDL-SPT complex and instead accumulates in stalled autophagosomes. Restoration of the defective autophagic flux by treatment with N-acetyl-L-leucine (NALL) (18) led to proper degradation of ORMDL and reversed sphingolipid accumulation. This work uncovered a previously unknown link between sphingolipid accumulation, ORMDL and autophagic defects present in NCP1 disease, in addition to providing further evidence and mechanistic insight for the beneficial role of NALL treatment for NPC1 disease that is currently awaiting FDA and EMA approval.

## Materials and Methods

### Cell culture and transient overexpression of ORMDL3

WT and NPC1 deleted HeLa cells (NPC1-KO) were a kind gift from Dr Wim Annaert (VIB center for Brain & Disease Research) (19). Nontargeting gRNAs or gRNAs targeting exon-2 (5′-AGGTACAATTGCGAATATTC-3′) and exon-4 (5′-AAAGAGTTACAATACTACGT-3′) inserted into pX330 plasmids were used with CRISPR/Cas9 genome editing technology to generate WT cells and NPC1-KO cells, respectively. Cells were cultured in Dulbecco’s modified Eagle’s medium (#11960-044, ThermoFisher) supplemented with fetal bovine serum (10% (v/v)), sodium pyruvate (1 mM) (#11360070, Gibco), glutamine (GlutaMAX 2 mM) (#35050061, Gibco) and penicillin-streptomycin (100 U/ml) (#15140122, Gibco) (complete-DMEM).

For overexpression of human ORMDL3, HeLa cells were grown to 70% confluence in 12-well plates or on coverslips for immunofluorescence, where indicated. The next day, the medium was replaced with a transfection mix containing 1 μg of ORMDL3-FLAG plasmid (#RC202279, NM_139280, Origene) or vector control (#PS100001, Origene), and 3 μl LipofectAMINE 2000 (#11668019, ThermoFisher) as previously described (20).

### Patient dermal fibroblasts

Dermal fibroblasts from a healthy control (GM08399) and fibroblasts from a patient with a homozygous mutation in NPC1I1061T (GM18453) were purchased from Coriell Institute for Medical Research and cultured in modified Eagle medium supplemented with 15% fetal bovine serum, 1 mM sodium pyruvate (#11360070, Gibco), 2 mM glutamine (#35050061, Gibco), and 100 U/ml penicillin-streptomycin (#15140122, Gibco).

### Western blotting

Cells were lysed in RIPA buffer (50 mM Tris-HCl; pH 7.4, 1 mM EDTA, 150 mM sodium chloride, 0.1% sodium dodecyl sulfate, 1% Triton X-100, 0.5% sodium deoxycholate) containing HALT protease/phosphatase inhibitor cocktail (#1861281, ThermoFisher). Equal amounts of protein were separated on a 10% or 12.5% SDS-PAGE gel and transferred to 0.2 μm pore-size nitrocellulose membrane (#1620112, BioRad) or PVDF membrane (#1620177, BioRad) with the PierceG2 Fast Blotter (#62287, ThermoFisher). Membranes were blocked for 1 h in TBST containing 5% BSA (#A7906, Sigma-Aldrich) and subsequently incubated overnight with continuous agitation at 4°C with the following primary antibodies diluted in TBST containing 5% BSA: anti-ORMDL3 (1:1,000; #ABN417, Sigma, RRID:AB_2943375), anti-NPC1 (1:1,000, #ab134113, Abcam, RRID:AB_2734695), anti-GAPDH (1:1,000, #5174, Cell Signaling Technology, RRID:AB_10622025), anti-SPTLC1 (1:1,000, #BD611305, BD Biosciences, RRID:AB_398831), anti-SPTLC2 (1:1,000, #51012-2-AP, Proteintech, RRID:AB_2195870), anti-LC3-I/II (1:1,000, #4108, Cell Signaling Technology, RRID:AB_2137703) and anti-beta-actin (1:1,000, #3700, Cell Signaling Technology, RRID:AB_2242334). Of note, although anti-ORMDL3 was generated against ORMDL3, due to the very high degree of homology between all three ORMDLs, the antibody also cross-reacts with ORMDL1 and ORMDL2. Membranes were then incubated for 1 h at room temperature in TBST containing 5% BSA and peroxidase-conjugated goat anti-rabbit secondary antibody (1:5,000; #NC9734651, FisherScientific) or peroxidase-conjugated goat anti-mouse secondary antibody (1:5,000; #NC9994806, FisherScientific) and protein bands visualized using chemiluminescent substrate (#34578, ThermoFisher). Immunoblot images were captured by Azure Imager C600 (Azure Biosystems, Inc) and analyzed with Image J. When possible, membranes were cut to enable probing of multiple proteins from the same gel. Densitometric quantification of immunoblot bands normalized to loading controls was by ImageJ.

### Quantification of sphingolipids by mass spectrometry

Cells were briefly washed with ice-cold PBS and scraped in 1 ml ice-cold HPLC-grade methanol. Internal standards were added, sphingolipids extracted, and long-chain bases and complex sphingolipids quantified by liquid chromatography electrospray ionization-tandem mass spectrometry (LC-ESI-MS/MS; API 5500 QTRAP; ABSciex) as previously described (21). Proteins were measured in identical technical replicates and sphingolipid levels expressed as pmol/mg protein.

### Immunofluorescence staining

Cells cultured on coverslips were briefly washed with ice-cold PBS, fixed for 15 min with paraformaldehyde (4%), and permeabilized for 3 min with Triton X-100 (0.1%) in PBS at room temperature. Cells were subsequently incubated for 45 min at room temperature in blocking buffer (5% FCS and 1% BSA in PBS). Cover slips were then removed from plates and incubated overnight at 4°C in a humidified chamber with the following primary antibodies diluted in blocking buffer: anti-calnexin (1:50, #2433, Cell Signaling Technology, RRID:AB_2243887), anti-FLAG (1:50, #F1804, Sigma-Aldrich, RRID:AB_262044), anti-p62 (1:50, #5144, Cell Signaling Technology, RRID:AB_10624872) and anti-LBPA (BMP) (1:50, #Z-PLBPA, Echelon Biosciences, RRID:AB_11129226). Cover slips were washed 3 times with ice-cold PBS and incubated for 1 h at room temperature in a dark, humidified chamber with the following secondary antibodies: anti-mouse Alexa Fluor 633 (1:100, #A21052, ThermoFisher, RRID:AB_2535719), anti-rabbit Alexa Fluor 488 (1:100, #A11008, ThermoFisher, RRID:AB_143165) and anti-mouse Alexa Fluor 555 (1:100, #A21422, ThermoFisher, RRID:AB_2535844). Cover slips were washed a further 3 times and mounted on to glass slides using VECTASHIELD vibrance antifade mounting medium with DAPI (H-1800).

### Confocal microscopy

An inverted Zeiss LSM880 confocal microscope equipped with a 63x PlanApo oil immersion lens (numerical aperture 1.4) was used to image cells. DAPI fluorescence was excited with a 405 nm laser diode, Alexa Fluor 488 fluorescence (and GFP) was excited using a 488 nm argon-ion laser, Alexa Fluor 555 (and RFP) was excited by a 561 nm diode-pumped solid-state laser and Alexa Fluor 633 was excited using a 633 nm helium-neon laser. The confocal pinhole was set to 1 Airy unit for an emission wavelength of 520 nm, and the pinhole was adjusted to the same size for all other emission wavelengths captured during the same experiment to achieve optical sectioning. DAPI emission was detected in the wavelengths 410–470 nm, Alexa Fluor 488 (and GFP) emission was detected at wavelengths 490–550 nm, Alexa Fluor 555 (and RFP) was detected at wavelengths 560–625 nm, and Alexa Fluor 633 was detected at emission wavelengths 640–700 nm. 16-bit images were captured for subsequent quantification. Analysis, quantification, and fluorescence distribution profiling of the immunofluorescence images were performed using ImageJ software. For quantification of LAMP1, p62, and BMP puncta, 16-bit images were equally threshold and quantification of size and number was automatically determined by particle analysis using ImageJ software.

### RFP-GFP-LC3B autophagy sensor

1 × 10^5^ HeLa cells cultured on glass coverslips were infected with 30 viral particles per cell of RFP-GFP-LC3 (#P36239, ThermoFisher) for 18 h. Confocal microscopic images taken for GFP and RFP were merged and the number of autophagosomes (GFP and RFP positive) and autolysosomes (RFP positive) were counted per cell. As fusion of the lysosome to the autophagosome leads to a drop in pH, quenching the acid-sensitive GFP, and therefore only autophagosomes are also positive for GFP. In some experiments, additional colocalization between GFP and ORMDL3-FLAG stained with Alexa Fluor 633 was used to determine the localization of ORMDL3-FLAG in stalled autophagosomes. For fluorescence distribution tracing, to further demonstrate the localization of ORMDL3-FLAG in autophagic vesicles, a straight line was drawn across a representative section of interest. The fluorescence intensities in the corresponding channels (Alexa Fluor 488 for green, Alexa Fluor 555 for red, and Alexa Fluor 633 for ORMDL-FLAG) were measured along the line. Results were normalized to the largest intensity observed in each channel and shown as line profiling graphs as described (22). Fluorescence intensity was expressed in arbitrary units.

### Co-immunoprecipitation

2 × 10^6^ HeLa cells in 100 mm culture dishes were trypsinized and resuspended in 1 ml of immunoprecipitation buffer (25 mM Tris-base; pH 7.4, 150 mM sodium chloride, 1 mM magnesium chloride, 1 mM calcium chloride, 15% glycerol) containing 1% glycol-diosgenin (GDN) (#GDN101, Anatrace) and HALT protease/phosphatase inhibitor cocktail (#1861281, ThermoFisher) for 1 h at 4°C with rotation. Lysed cells were centrifuged at 23,000 g for 15 min at 4°C. The supernatant was collected and 40 μl was added to 10 μl of 5X denaturing Laemmli buffer and heated at 57°C for 1 h to be used as the input. The remaining supernatant was pre-cleared using IgG isotype control antibody (#30000-0AP, Proteintech, RRID:AB_2819035) (1 μl antibody to 200 μl supernatant) and protein A/G Sepharose beads (#SC-2003, Santa Cruz Biotechnology) (40 μl per sample) for 1 h at 4°C with rotation. Samples were centrifuged at 23,000 g for 2 min at 4°C, and the supernatants were incubated overnight at 4°C with rotation with either IgG isotype control antibody (#30000-0AP, Proteintech, RRID:AB_2819035) or anti-SPTLC2 antibody (1:1,000, #51012-2-AP, Proteintech, RRID:AB_2195870) (200 μl supernatant:1 μl antibody). Following centrifugation at 23,000 g for 20 min at 4°C, supernatants were incubated with protein A/G Sepharose beads (#SC-2003, Santa Cruz Biotechnology) (20 μl per sample) for 1 h at 4°C with rotation. Beads were then centrifuged at 23,000 g for 2 min at 4°C and washed with 0.5 ml immunoprecipitation buffer containing 0.1% GDN and HALT protease/phosphatase inhibitor cocktail by rotation at 4°C for 10 min. Beads were again centrifuged at 23,000 g for 2 min at 4°C before 30 μl of 2X denaturing Laemmli buffer was added and heated at 57°C for 1 h. Equal volumes of protein lysates of input and immunoprecipitates were analyzed by Western blotting.

### Statistics

All data are from biological triplicates and are representative of at least three independent experiments. For each experiment, N denotes biological replicates, and n is the number of technical replicates. For immunofluorescence experiments analysis was performed using 10 cells from each of the 3 biological replicates (30 replicates total). Statistical significance was calculated with a two-tailed Student’s *t* test for the comparison of two groups, or with ANOVA followed by post hoc tests for multiple comparisons using GraphPad Prism 8 software. For all experiments, the normality of the data from each group was first checked using the Shapiro-Wilk test. The designations for significance levels are ∗*P* ≤ 0.05, ∗∗*P* ≤ 0.01, ∗∗∗*P* ≤ 0.001 and ∗∗∗∗*P* ≤ 0.0001.

## Results

### Deletion of NPC1 enhances de novo sphingolipid biosynthesis yet increases ORMDL levels

**Fig. 1:**
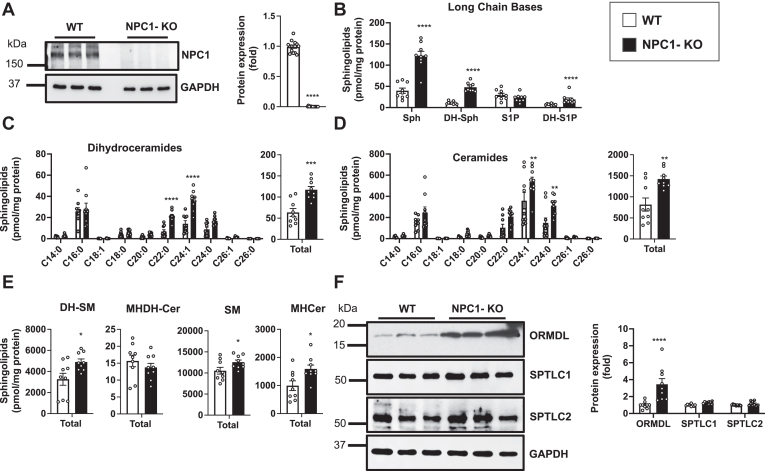
Deletion of NPC1 enhances de novo sphingolipid biosynthesis, yet increases ORMDL levels. A: NPC1 levels in WT and NPC1-KO HeLa cells were determined by immunoblot analysis with anti-NPC1 antibody. GAPDH was used as a loading control. Blots were quantitated by densitometry (N = 5, n = 3). B–E: Sphingolipids were extracted from WT and NPC1-KO HeLa cells and levels of sphingosine (Sph), dihydrosphingosine (DH-Sph), sphingosine-1-phosphate (S1P), dihydrosphingosine-1-phosphate (DH-S1P), ceramide (Cer), dihydroceramide (DH-Cer), sphingomyelin (SM) dihydrosphingomyelin (DH-SM) monohexosylceramide (MHCer) and monohexosyldihydroceramide, (MHDH-Cer) were determined by LC-ESI-MS/MS. Different chain-length species of dihydroceramide (C) and ceramide (D) are shown; numbers denote the acyl chain lengths followed by the number of double bonds in the fatty acid. (N = 3, n = 3). F: Protein levels of ORMDLs, SPTLC1 and SPTLC2 in WT and NPC1-KO cells were determined by immunoblotting. GAPDH was used as a loading control. Blots were quantitated by densitometry (N = 3, n = 3). Data are mean ± SEM. ∗*P* ≤ 0.05, ∗∗*P* ≤ 0.01, ∗∗∗*P* ≤ 0.001 and, ∗∗∗∗*P* ≤ 0.0001 compared to WT. Unpaired two-tailed Student’s *t* test for total sphingolipid amount and two-way analysis of variance test followed by Bonferroni's multiple comparison test for sphingolipid species.

Consistent with earlier research demonstrating that in addition to cholesterol, sphingolipids also accumulate in NPC1 patients, animal models of NPC1, as well as in NPC1 mutant primary fibroblasts (5, 6, 19, 23), we also found that levels of ceramide and complex sphingolipids were elevated in NPC1-deleted HeLa cells (NPC1-KO) (Fig. 1A–E). Additionally, the level of sphingosine was increased by almost 3-fold in NPC1-KO cells (Fig. 1B). This correlates with evidence that NPC1 exports sphingosine from the lysosome to the limiting membrane of the lysosome for transport at lysosome–ER contact sites (11). Moreover, dihydrosphingosine and dihydroceramide, two intermediates generated early in the sphingolipid biosynthetic pathway exclusively through de novo biosynthesis, were increased by 4.3-fold and 1.9-fold, respectively (Fig. 1B, C). Most pronounced were the increases of very-long-chain N-acyl chain C20:0 and C24:1 dihydroceramide and ceramide species (Fig. 1C, D). The increase in dihydroceramide led to increased levels of dihydrosphingomyelin but not monohexosyldihydroceramide in these cells (Fig. 1E).

As the ORMDL-SPT complex regulates de novo sphingolipid biosynthesis (14, 15, 16), we sought to assess the expression of individual subunits of this complex. No major changes were observed in expression of SPTLC1 or SPTLC2, the essential subunits of the SPT, which catalyze the first and rate-limiting step in the de novo biosynthesis of ceramide. Surprisingly, despite the increased de novo sphingolipid biosynthesis in NPC1-KO cells, immunoblotting with an antibody that recognizes all three ORMDL isoforms showed that there were increased rather than decreased levels of ORMDL proteins (Fig. 1F), the negative regulators of SPT activity (14, 15, 16). Although treatment of WT cells with sphingosine also increased levels of ORMDL proteins, this effect was much smaller compared to NPC1-deleted cells (Supplemental Fig. S1 and Fig. 1F).

### Inhibition of the NPC1 sterol binding domain leads to increased de novo sphingolipid biosynthesis and ORMDL expression

**Fig. 2:**
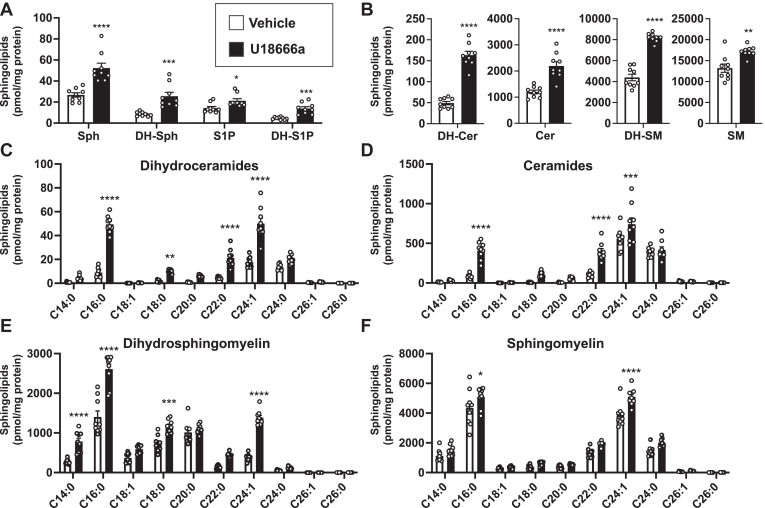
Pharmacological inhibition of NPC1 increased de novo sphingolipid biosynthesis. WT HeLa cells were treated with vehicle or U18666a (5 μM). A, B: After 24 h, sphingolipids were extracted and levels of sphingosine (Sph), dihydrosphingosine (DH-Sph), sphingosine-1-phosphate (S1P), dihydrosphingosine-1-phosphate (DH-S1P), ceramide (Cer), dihydroceramide (DH-Cer), sphingomyelin (SM), dihydrosphingomyelin (DH-SM) were determined by LC-ESI-MS/MS. Different chain-length species of dihydroceramide (C), ceramide (D) dihydrosphingomyelin (E) and sphingomyelin (F) are shown; numbers denote the acyl chain lengths followed by the number of double bonds in the fatty acid. (N = 3, n = 3). Data are means ± SEM. ∗*P* ≤ 0.05, ∗∗*P* ≤ 0.01, ∗∗∗*P* ≤ 0.001 and ∗∗∗∗*P* ≤ 0.0001 compared to vehicle treated cells. Unpaired two-tailed Student’s *t* test for total sphingolipid amount and two-way analysis of variance test followed by Bonferroni's multiple comparison test for sphingolipid species.

**Fig. 3:**
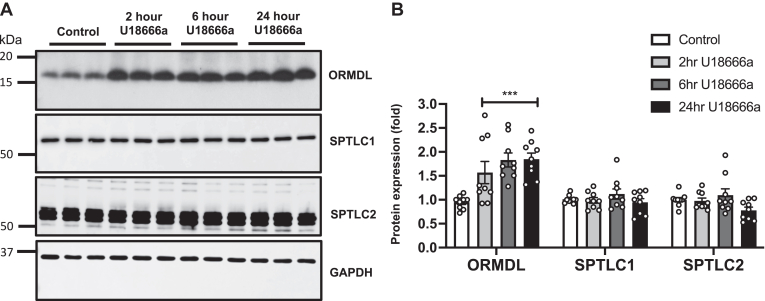
NPC1 inhibitor increases ORMDL levels. HeLa cells were treated with vehicle or U18666a (5 μM) for the indicated time. A: Protein levels of ORMDLs, SPTLC1 and SPTLC2 in cell lysates were determined by immunoblotting. GAPDH was used as a loading control. B: Blots were quantitated by densitometry (N = 3, n = 3). Data are means ± SEM. ∗∗∗*P* ≤ 0.001 compared to cells treated with vehicle. Two-way analysis of variance test followed by Tukey's multiple comparison test.

Because of the unexpected finding that ORMDL levels were increased concomitantly with increased de novo sphingolipid biosynthesis in NPC1-KO cells, we used U18666A, a cationic amphiphile that binds the sterol-sensing domain of NPC1 (24) and blocks the movement of cholesterol out of lysosomes (25, 26) thereby mimicking the NPC1 deletion phenotype. As expected, treatment of HeLa cells with U18666a markedly increased all sphingolipids, including dihydrosphingosine, dihydrosphingosine-1-phosphate, dihydroceramides, and dihydrosphingomyelins (Fig. 2A, B). There were large increases in almost all dihydroceramides and ceramides species especially C16:0, C22:0 and C24:1 long-chain and very-long-chain N-acyl chain species (Fig. 2C, D). Likewise, significant increases were found in C16:0, and C24:1 dihydrosphingomyelin and sphingomyelin (Fig. 2E, F). Pharmacological inhibition of NPC1 also increased ORMDL levels within 2 h, without affecting levels of SPTLC1 or SPTLC2 (Fig. 3A, B).

### Deletion of NPC1 decreased ORMDLs in the SPT complex

**Fig. 4:**
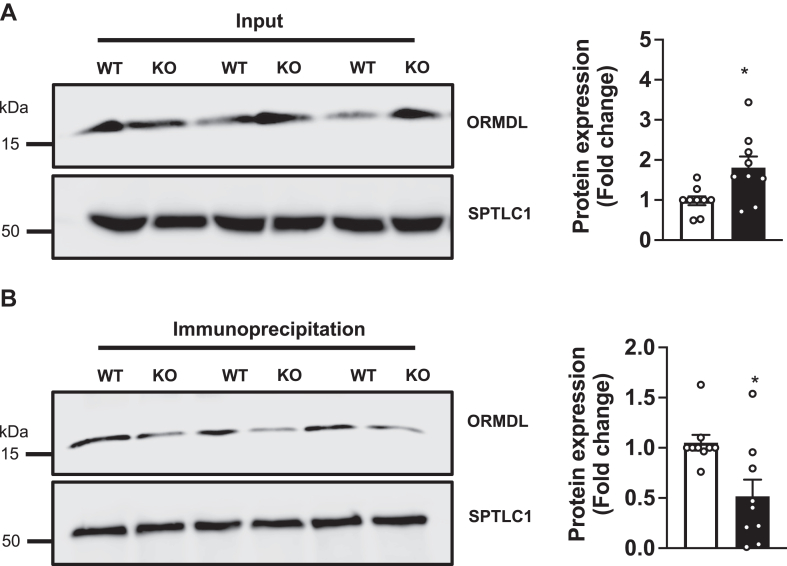
Reduced ORMDL in complex with SPT in NPC1 deleted cells. SPTLC2 was immunoprecipitated from WT and NPC1-KO HeLa cells. Protein levels of SPTLC1 and ORMDLs were detected in inputs (A) and immunoprecipitates (B) and densitometric analyses of ORMDL blots are shown in the right panels normalized to SPTLC1. Data are fold increases compared to WT and are the means ± SEM (N = 9). ∗*P* ≤ 0.05 compared to WT cells. Unpaired two-tailed Student’s *t* test.

Recent cryo-EM studies clearly demonstrate that the ORMDL proteins are in complex with SPT, sense elevated ceramide levels, and inhibit SPT enzymatic activity, effectively preventing the incorporation of acyl-CoA into newly synthesized sphingolipid substrate dihydrosphingosine, thereby inhibiting sphingolipid biosynthesis (14, 15, 16, 27). Therefore, we next examined the effect of NPC1 deletion on interaction of ORMDL with SPTLC1 and SPTLC2 heterodimer complex. SPTLC2 was immunoprecipitated from digitonin-solubilized membrane fractions of WT and NPC1-KO cells, and input and eluate fractions were analyzed by immunoblotting (Fig. 4A, B). The obligatory SPTLC1 subunit of the SPT complex was used as a loading control and to confirm retention of ORMDL with SPTLC2 and SPTLC1 in the ORMDL-SPT complex. Nevertheless, there was a significant reduction in the amount of ORMDL bound to SPTLC2 and thus in the SPT complex (Fig. 4B). This data suggests that although there is an increase in ORMDL expression in NPC1-KO cells, the protein is not efficiently bound in a complex with SPT and therefore the catalytic activity of SPT remains high despite elevated ceramide levels.

### Colocalization of ORMDL with the selective autophagy receptor p62 in NPC1 deficient cells

**Fig. 5:**
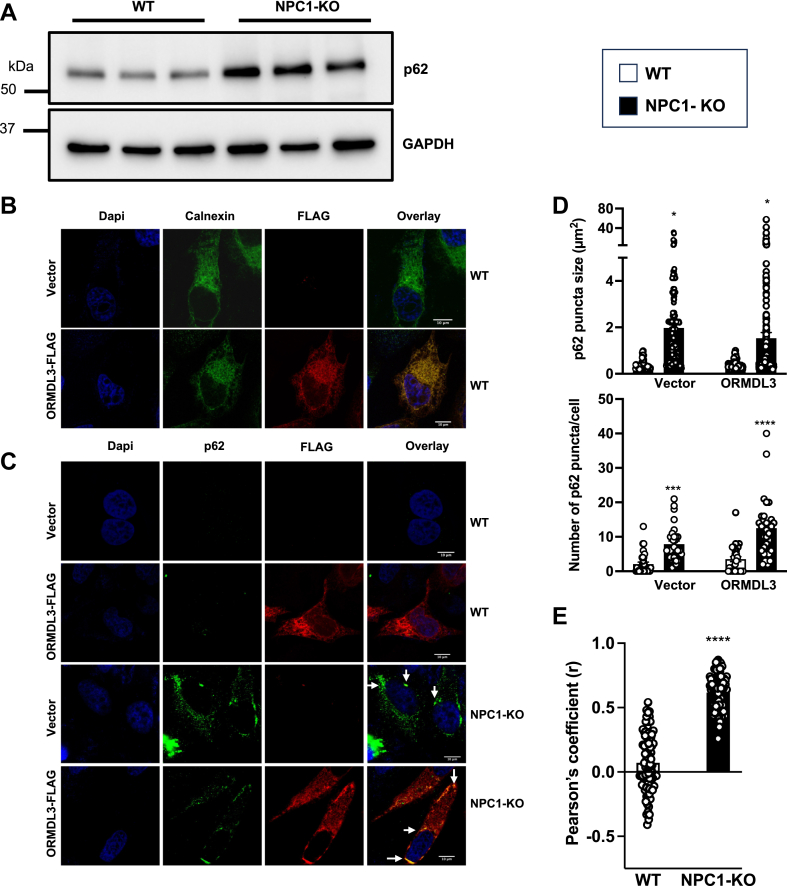
ORMDL colocalizes with p62-positive aggregates in NPC1 deleted cells. A: Western blots of p62 in WT and NPC1-KO HeLa cells. GAPDH was used as a loading control (N = 3). B: Representative confocal microscopic images of WT cells transiently expressing vector or ORMDL3-FLAG (red), co-stained with anti-calnexin antibody to the ER (green), and DAPI to stain nuclei (blue). C: Representative confocal microscopic images of WT and NPC1-KO cells transiently expressing vector or ORMDL3-FLAG (red), co-stained with anti-p62 antibody to visualize autophagosomes (green), and DAPI to stain nuclei (blue). D: Accumulation of autophagosomes (arrows), a feature of NPC1 disease, was quantified by measuring the size and number of p62 puncta per cell automatically enumerated with Image J. E: Pearson’s correlation coefficient of p62 and ORMDL3 co-localization in WT and NPC1-KO cells (N = 3, n = 10 cells analyzed for each group). Data are the means ± SEM. ∗*P* ≤ 0.05, ∗∗∗*P* ≤ 0.001 and ∗∗∗∗*P* ≤ 0.0001 compared to WT. D: One-way analysis of variance test followed by Tukey's multiple comparison test. E: Unpaired two-tailed Student’s *t* test.

Although the level of ORMDL is high in NPC1-KO cells, we observed that the amount of protein that is in complex with SPT is reduced. Thus, we asked whether ORMDL was localized to subcellular compartments other than in complex with SPT at the ER. In this regard, it has previously been shown that increased intracellular-free cholesterol-induced ORMDL redistribution from the ER to cytoplasmic p62/SQSTM1-positive autophagosomes (28). As commercial antibodies are not available for the detection of ORMDL by immunofluorescence, we utilized C-terminal tagged ORMDL3 (ORMDL3-FLAG) that can be detected using antibodies against the FLAG epitope. ORMDL3 was chosen as it has recently been shown to be the most responsive ORMDL isoform to changes in ceramide levels (16) and suppressing SPT activity (29, 30). In agreement with previous reports (31, 32), NPC1-KO cells had markedly increased p62 (Fig. 5A), a ubiquitin-binding scaffold protein that targets specific cargoes for autophagy and accumulates when autophagy is inhibited. ORMDL3-FLAG transiently overexpressed in WT cells was observed in a classical reticular ER pattern colocalized with calnexin (Fig. 5B) with little or no colocalization with p62 (Fig. 5C). Deletion of NPC1 also increased p62 aggregation, determined by puncta size and number, consistent with autophagosome accumulation (Fig. 5C, D). In addition, in NPC1-KO cells ORMDL3-FLAG formed aggregates that colocalized with enlarged p62 puncta (Fig. 5C–E), a feature of impaired autophagic flux.

### Impaired autophagosome maturation due to NPC1 deletion or pharmacological inhibition retards autophagic ORMDL cargo clearance

As our data suggest that ORMDL3 remains stalled in immature autophagosomes in NPC1 deleted cells, we sought to analyze this process in more detail using a tandem-fluorescent-tagged–mRFP-GFP-LC3 sensor (33). This chimeric protein expressing LC3B (LC3-II) tagged with acid-sensitive green fluorescent protein (GFP) and acid-insensitive monomeric red fluorescent protein (mRFP) enables the visualization of autophagosomes that emit both GFP and RFP signals, and autolysosomes emitting solely RFP as the pH-sensitive GFP signal is quenched in acidic lysosomes (33).

**Fig. 6:**
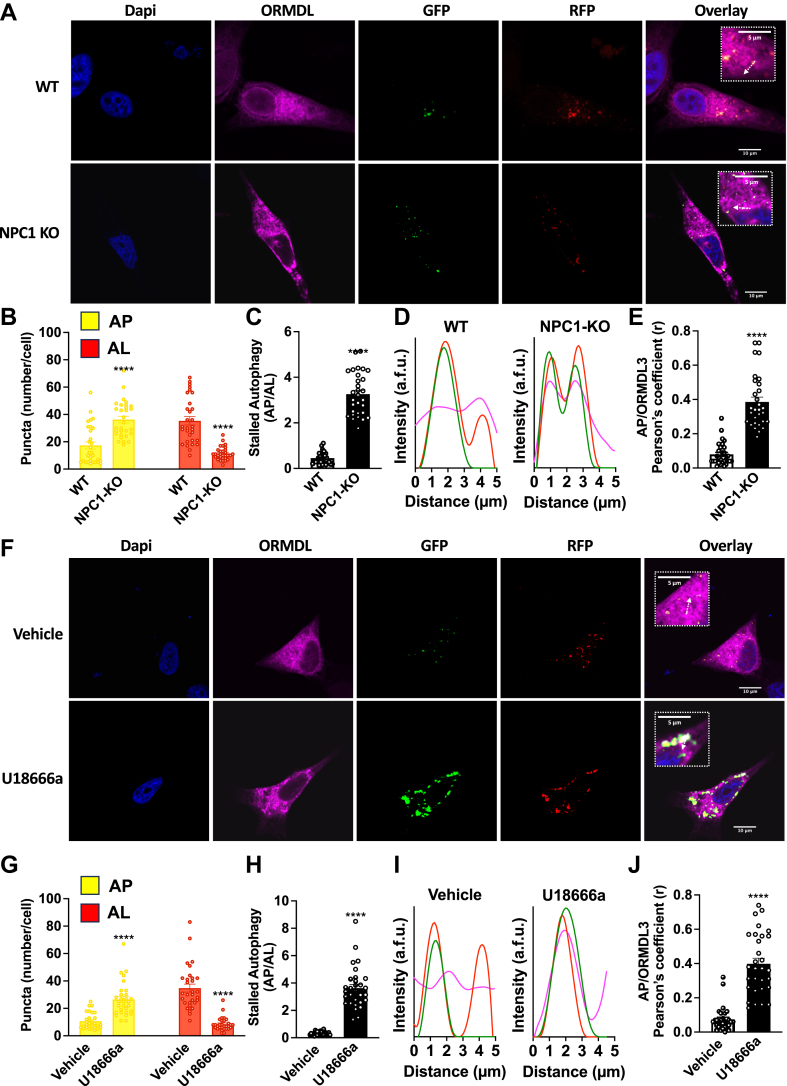
ORMDL accumulates in autophagosomes due to pharmacological inhibition or deletion of NPC1. A and F: Representative confocal microscopic images of WT and NPC1-KO cells, or WT cells treated with vehicle or U18666a (5 μM) for 24 h, transiently overexpressing ORMDL3-FLAG (Magenta) and RFP-GFP-LC3B tandem sensor to label autophagosomes (AP) expressing GFP and RFP (yellow in overlay), and autolysosomes (AL) expressing only RFP (red in overlay). Cells were co-stained with DAPI to visualize nuclei. B and G: Autophagosomes and autolysosomes were quantified by measuring the number of yellow and red puncta per cell in the overlayed image. C and H: Stalled autophagy was determined from the ratio of autophagosomes to autolysosomes per cell. D and I: Pixel intensity tracing along the indicated white line displayed localization of autophagosomes (red and green lines) and ORMDL3-FLAG (magenta line). E and J: Pearson’s correlation coefficient of colocalization between ORMDL-FLAG and GFP (autophagosomes). (N = 3, n = 10 cells for each group). Data are the means ± SEM. ∗∗∗∗*P* ≤ 0.0001 compared to WT. B and G: One-way analysis of variance test followed by Tukey's multiple comparison test. (C, E, H and J) Unpaired two-tailed Student’s *t* test.

As expected, there were more autolysosomes than autophagosomes in WT (Fig. 6A, B) or vehicle-treated cells (Fig. 6F, G), and neither organelle correlated with localization of ORMDL3-FLAG (Fig. 6D, E, I, J). In contrast, NCP1- KO cells (Fig. 6A) or cells treated with the pharmacological NPC1 inhibitor U18666a (Fig. 6F) displayed increased number of autophagosomes and decreased abundance of autolysosomes (Fig. 6B, G), indicative of stalled autophagy (Fig. 6C, H). Moreover, ORMDL3-FLAG that formed aggregates in NCP1-KO cells and cells treated with U18666a significantly colocalized with autophagosomes (GFP) quantified by Pearson’s correlation coefficient and fluorescence distribution tracing (Fig. 6D, E, I, J). Together, these data suggest that ORMDL is sent for autophagic degradation but remains in immature autophagosomes due to defective autophagy in NPC1 disease.

### Restoration of autophagic flux with N-acetyl-L-leucine in NPC1-deleted cells decreases ORMDL accumulation in autophagosomes and reduces the accumulation of sphingolipids and their de novo biosynthesis

**Fig. 7:**
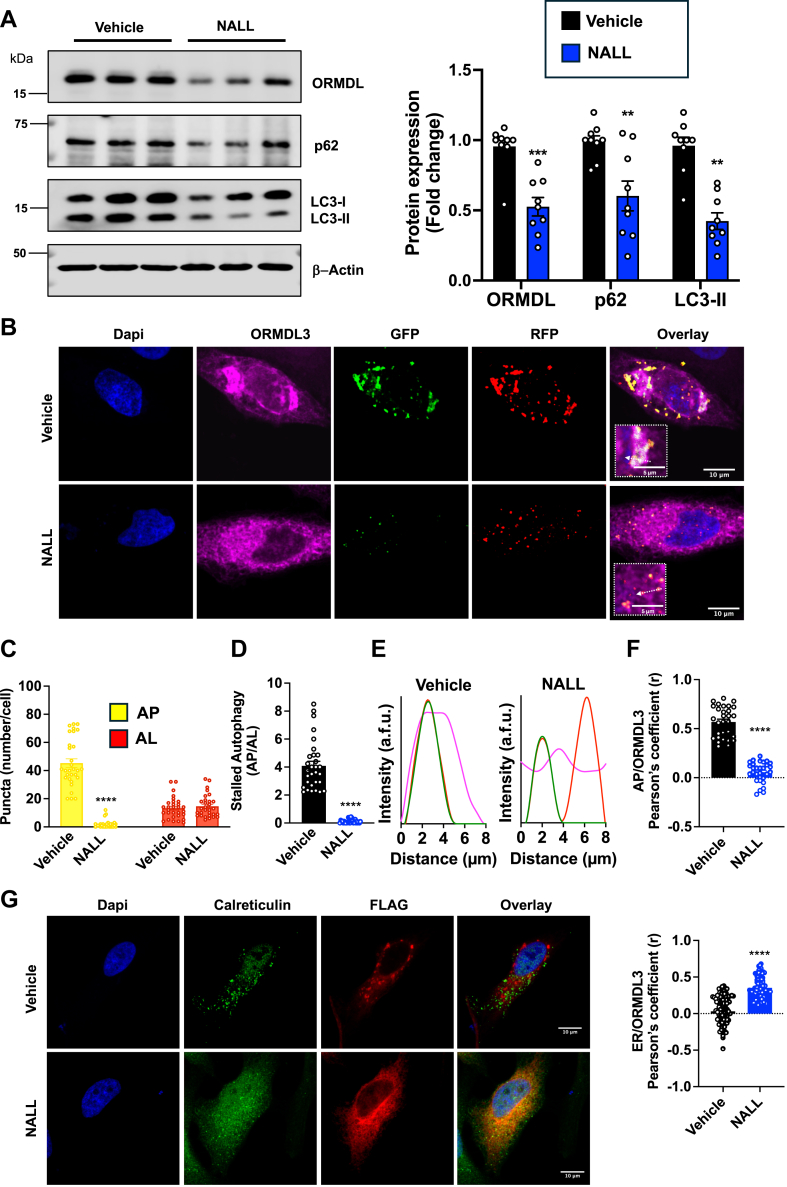
N-acetyl-L-leucine restores autophagic flux in NPC1 KO cells and reduces ORMDL aggregation in autophagosomes. A: Protein levels of ORMDL, p62 and LC3-II were determined in NPC1-KO cells treated with vehicle or N-acetyl-L-leucine (NALL) (5 mM) for 24 h by immunoblotting. β-actin was used as a loading control. Blots were quantitated by densitometry (N = 3, n = 3). B–F: Representative confocal microscopic images of NPC1 KO cells transiently overexpressing ORMDL3-FLAG (Magenta) and transduced with RFP-GFP-LC3B tandem sensor to label autophagosomes (AP) expressing GFP and RFP (yellow in overlay), and autolysosomes (AL) expressing only RFP (red in overlay), treated with vehicle or NALL (5 μM) for 24 h. Cells were co-stained with DAPI to visualize nuclei. C: Autophagosomes and autolysosomes were quantified per cell by measuring the number of yellow and red puncta in the overlayed image. D: Stalled autophagy was determined from the ratio of autophagosomes to autolysosomes per cell. E: Pixel intensity tracing along the indicated white line displayed localization of autophagosomes (red and green lines) and ORMDL3-FLAG (magenta line). F: Pearson’s correlation coefficient of colocalization between ORMDL-FLAG and GFP (autophagosomes). (N = 3, n = 10 cells for each group). G: NPC1-KO cells overexpressing ORMDL3-FLAG were treated with vehicle or NALL (5 μM) for 24 h. Representative confocal microscopic images show immunofluorescence staining of ORMDL3-FLAG (red) and the ER marker calreticulin (green), and nuclei co-stained with DAPI. Pearson’s correlation coefficient of colocalization between ORMDL3 and the ER marker calreticulin. (N = 3, n = 10 cells for each group). Data are the means ± SEM. ∗∗*P* ≤ 0.01, ∗∗∗*P* ≤ 0.001 and ∗∗∗∗*P* ≤ 0.0001 compared to vehicle treated. C: One-way analysis of variance test followed by Tukey's multiple comparison test. (A, D, F and G) Unpaired two-tailed Student’s *t* test.

Following our observation that ORMDL accumulates in stalled autophagosomes, it was of interest to examine whether enhancement of autophagic flux would restore ORMDL degradation and reverse ORMDL accumulation in stalled autophagic vesicles. To this end, we selected to use N-acetyl-L-leucine (NALL). NALL has been recently shown to improve neurological impairments and slow disease progression in *Npc1*^*−/−*^ mice (34) and NPC patients (35, 36). Moreover, NALL treatment restores autophagic flux by alleviating p62 and LC3-II build-up in mouse models of traumatic brain injury to prevent cortical cell death and cognitive decline (18). Indeed, treatment with NALL for 24 h led to a reduction in the levels of the p62 and LC3-II accumulated in NPC1-KO cells (Fig. 7A) In addition, there was a striking reduction in levels of ORMDL in these cells (Fig. 7A). To examine whether induction of autophagy by NALL contributes to enhanced degradation of ORMDL accumulated in autophagosomes of NPC1-KO cells, we used bafilomycin A_1_ (bafA_1_), which disrupts autophagic flux by inhibiting V-ATPase-dependent lysosomal acidification. Treatment of these cells with bafA1 reversed the effect of NALL and markedly increased levels LC3-II and p62, and ORMDL to a lesser extent, by inhibiting their degradation (Supplemental Fig. S2).

**Fig. 8:**
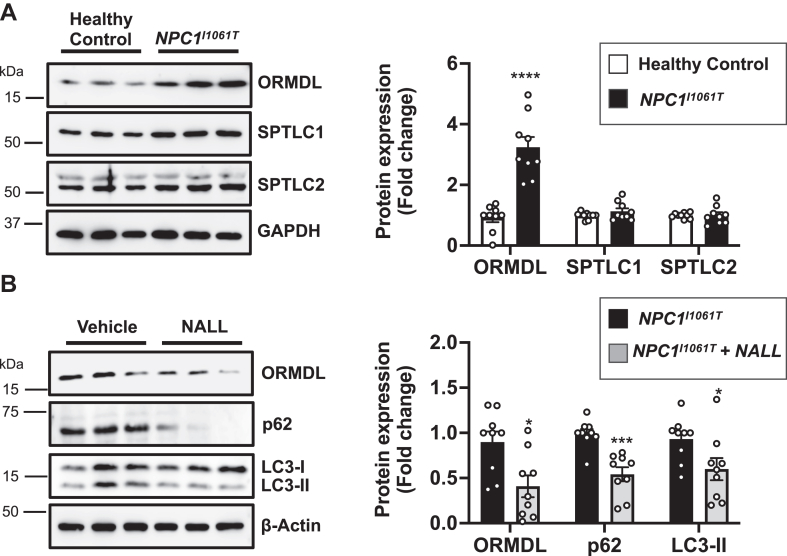
ORMDL levels are increased in *NPC1*^*I1061T*^ mutant fibroblasts and treatment with N-acetyl-L-leucine reduces accumulation of ORMDLs, p62, and LC3-II. A: Protein levels of ORMDLs, SPTLC1 and SPTLC2 in *NPC1*^*I1061T*^ mutant fibroblasts and healthy control fibroblasts were determined by immunoblotting. GAPDH was used as a loading control. B: Protein levels of ORMDL, p62 and LC3-II were determined in *NPC1*^*I1061T*^ mutant fibroblasts treated with vehicle or N-acetyl-L-leucine (NALL) (5 mM) for 24 h by immunoblotting. β-actin was used as a loading control. (A and B) Blots were quantitated by densitometry (N = 3, n = 3). Data are mean ± SEM. ∗*P* ≤ 0.05, ∗∗∗*P* ≤ 0.001 and ∗∗∗∗*P* ≤ 0.0001 compared to healthy control. Unpaired two-tailed Student’s *t* test.

We next sought to extend our findings to human primary NPC1 mutant fibroblasts, in which both alleles carry the *I1061T* mutation, the most common mutation observed in NPC1 patients that represents 15%–20% of all disease alleles (37). As in NPC1-KO cells (Fig. 1F), levels of ORMDL but not SPTLC1 or SPTLC2, were increased in *NPC1*^*I1061T*^ mutant fibroblasts compared to healthy controls (Fig. 8A). Likewise, treatment of *NPC1*^*I1061T*^ mutant fibroblasts with NALL also reduced accumulation of ORMDLs, p62, and LC3-II (Fig. 8B).

To further confirm that stimulating autophagy normalized ORMDL degradation in autophagosomes, NPC1-KO cells expressing ORMDL3-FLAG were transduced with RFP-GFP-LC3B tandem sensor, in which ORMDL3 aggregates in autophagosomes (Fig. 5C–E and Fig. 6A–E). Treatment with NALL for 24 h led to an almost complete abolishment of visible autophagosomes in these NPC1-KO cells, while retaining the formation of autolysosomes (Fig. 7B, C), indicating the restoration of autophagic flux (Fig. 7D). Importantly, ORMDL3 aggregates were no longer visible in these NALL treated NPC1-KO cells and were not colocalized with GFP fluorescing autophagosomes (Fig. 7B, E, F). Rather, NALL treatment increased colocalization of ORMDL3 with the ER marker calreticulin (Fig. 7G). These finding suggest that restoring clearance of autophagic cargo prevents ORMDL accumulation in autophagosomes and retains its ER localization.

**Fig. 9:**
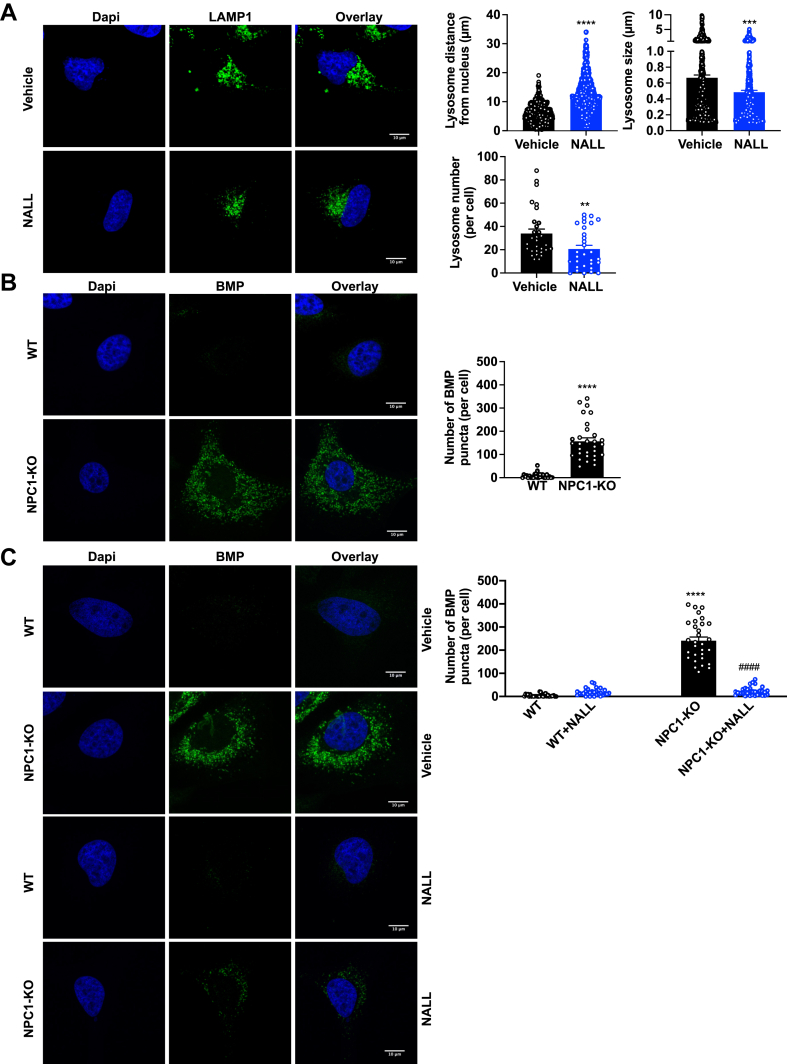
N-acetyl-L-leucine reduces clustered lysosomes in perinuclear regions, and bis(monoacylglycero)phosphate accumulation in NPC1 deleted cells. A: NPC1-KO cells were treated with vehicle or NALL (5 μM) for 24 h. Representative confocal images of LAMP1 immunofluorescence staining (green) and nuclei co-stained with DAPI. Quantification of the distance of lysosomes from nuclei as well as lysosome size and numbers per cell were enumerated with ImageJ. B and C: Representative confocal microscopic images of WT or NPC1-KO cells treated without (B) or with vehicle or NALL (5 μM) for 24 h (C) and stained with anti-BMP antibodies. Quantification of numbers of BMP puncta per cell. (N = 3, n = 10 cells for each group). Data are mean ± SEM. ∗∗*P* ≤ 0.01, ∗∗∗*P* ≤ 0.001 and ∗∗∗∗*P* ≤ 0.0001 compared to WT or vehicle treated. ^####^*P* ≤ 0.0001 compared to vehicle treated of NPC1-KO. A and B: Unpaired two-tailed Student’s *t* test. C: One-way analysis of variance test followed by Tukey's multiple comparison test.

NPC1 deletion disrupts lysosome distribution leading to perinuclear accumulation of lysosomes and lysosomal enlargement (38). Immunofluorescent staining of LAMP1, a major lysosomal marker, revealed reductions of clustered lysosomes in perinuclear regions and decreased lysosomal size and numbers following treatment of NPC1-KO cells with NALL (Fig. 9A).

**Fig. 10:**
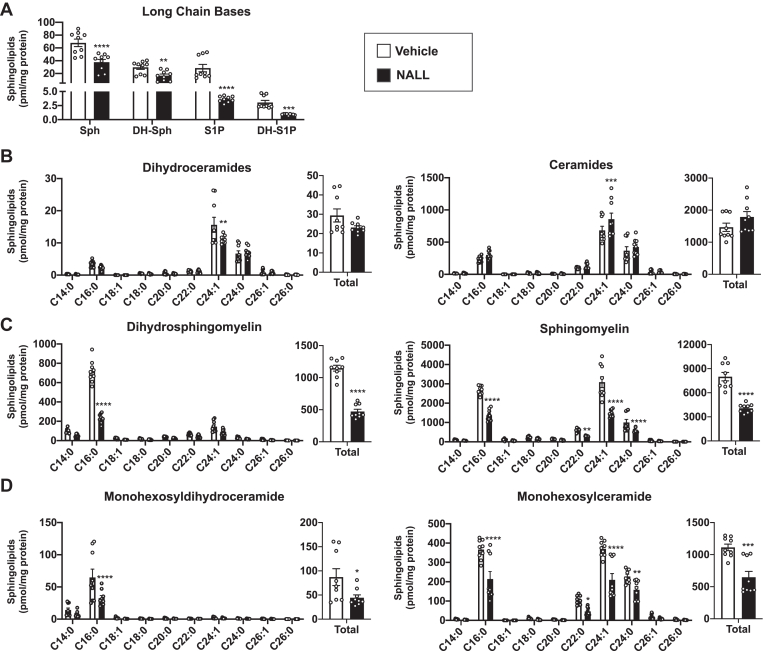
N-acetyl-L-leucine reduces de novo biosynthesis and accumulation of sphingolipids. Sphingolipids were extracted from NPC1-KO cells treated with vehicle or NALL (5 mM) for 24 h which restore autophagic flux. Levels of (A) sphingosine (Sph), dihydrosphingosine (DH-Sph), sphingosine-1-phosphate (S1P), dihydrosphingosine-1-phosphate (DH-S1P), (B) dihydroceramide, ceramide (C) dihydrosphingomyelin, sphingomyelin, (D) monohexosyldihydroceramide and monohexosylceramide were determined by LC-ESI-MS/MS. Different chain-length species of sphingolipid as well as total level are shown; numbers denote the acyl chain lengths followed by the number of double bonds in the fatty acid. (N = 3, n = 3). Data are means ± SEM. ∗∗*P* ≤ 0.01, ∗∗∗*P* ≤ 0.001 and ∗∗∗∗*P* ≤ 0.0001 compared to cell treated with vehicle. Unpaired two-tailed Student’s *t* test for total sphingolipid amount and two-way analysis of variance test followed by Bonferroni's multiple comparison test for sphingolipid species.

NALL has been shown to reduce some lipid species in the brain and non-neuronal tissues of *NPC1*^*−/−*^ mice (34). Therefore, we next also tested whether the restoration of ORMDL localization simultaneously led to changes in sphingolipids and in bis(monoacylglycero)phosphate (BMP; also known as lysobisphosphatidic acid), both lipids known to be increased due to NPC1 deficiency (39). As expected NPC1 deletion increased BMP staining (Fig. 9C) that was suppressed by NALL (Fig. 9B). NALL treatment of NPC1-KO cells also induced a striking reduction in the long-chain sphingoid bases, sphingosine, S1P, and importantly in dihydrosphingosine, consistent with a reduction in de novo sphingolipid biosynthesis (Fig. 10A). NALL had only minor effects on total levels of dihydroceramide with significant reduction only in C24:1 species. Surprisingly, this treatment did not affect levels of total ceramides (Fig. 10B). Nevertheless, NALL significantly reduced C16:0 dihydrosphingomyelin and monohexosyldihydroceramide, the major species of these classes (Fig. 10C, D). In addition, NALL caused a robust reduction in many of the long- and very-long-chain sphingomyelin and monohexosylceramides that were especially evident in C16:0, C22:0, C24:1, and C24:0 species (Fig. 10C, D). Collectively, these data suggest that restoring the autophagic flux by NALL is sufficient to degrade ORMDL stalled in autophagosomes leading to a reduction in de novo sphingolipid biosynthesis and decreasing the accumulation of complex sphingolipids observed in NPC1-KO cells.

## Discussion

While extensive research has clarified the causal link between NPC1 mutations and the build-up of cholesterol and sphingolipids within lysosomes (2, 3, 4, 5, 6, 7, 8), surprisingly until now, little was known about the mechanisms driving the accumulation of dihydrosphingosine, the first sphingolipid synthesized in the de novo biosynthetic pathway. Unraveling the interplay between NPC1 deficiency and the increase in de novo sphingolipid biosynthesis could offer insights into the pathophysiology of NPC1 disease and potentially reveal new therapeutic avenues aimed at mitigating sphingolipids accumulation and their detrimental effects on cellular function contributing to neurological symptoms associated with this disease.

We found that although deletion of NPC1 enhanced de novo sphingolipid biosynthesis, in contrast to our expectation, there were no changes in levels of SPTLC1 or SPTLC2, the essential subunits of the SPT. Surprisingly, there were increased rather than decreased levels of ORMDL, the negative regulators of SPT activity (14, 15, 16). Similar results were observed by acute pharmacological NPC1 inhibition using U18666A, which binds to the sterol-sensing domain of NPC1 and block the intracellular trafficking of cholesterol (25). Although U18666a also targets other sterol homeostatic proteins (40) in addition to NPC1, and may have some lysosomotropic side effects, it should be noted that like NPC1 deletion, levels of ORMDL, but not SPTLC1 or SPTLC2, were also significantly increased in patient fibroblasts carrying the most prevalent mutation, NPC1(I1061T), which dramatically decreases NPC1 protein levels (37, 41). Even though there is an increase in ORMDL levels in NPC1 deficient cells, the protein is not efficiently bound in a complex with SPT. As a result, despite elevated ceramide levels, the catalytic activity of SPT is not inhibited and remains high. This data explains the disparity between elevated level of ORMDL and increased dihydrosphingosine. This is analogous to other disease pathologies such as hereditary sensory and autonomic neuropathy type 1, whereby there is reduced ORMDL in complex with SPT resulting in elevated SPT activity and increased de novo sphingolipids (42).

It is also possible that the observed elevated de novo sphingolipid biosynthesis may be due to the lack of ceramide at the ER that is sensed by ORMDL. It was elegantly demonstrated that when ceramide level at the ER increases, it binds to ORMDL in complex with SPT locking the N-terminus of ORMDL in a conformation that blocks entry of acyl-CoA into the pocket of SPT to inhibit de novo sphingolipid synthesis (16, 27). In the absence of NPC1, sphingosine that accumulates in LE/LY is unable to exit them to reach the ER for recycling into ceramide (11, 26). Therefore, the elevated level of ceramide cannot be detected by ORMDL to suppress SPT activity. In this regard, although treatment with sphingosine also increased levels of ORMDLs, this effect was less significant compared to NPC1-deletion. Sphingosine can be metabolized in cells to ceramide or S1P and both have been implicated in the regulation of ORMDL (16, 27, 43), thus complicating interpretations.

In the absence of functional NPC1, we observed that excess ORMDL co-localizes with p62 aggregates and accumulates in stalled autophagosomes resulting from impaired autophagic flux and defective cargo clearance. Impaired autophagy flux in NPC1 disease has been attributed to defective amphisome formation through a failure in SNARE trafficking (31) or prevention of autophagosome–lysosome fusion (44). Our data is consistent with the observation that the autophagic complications in NPC1 diseased cells arise from defects in autophagosome maturation and not defective lysosomal proteolytic function (31). Impaired flux has been shown to contribute to the lysosomal storage of cholesterol and sphingolipids (8, 31, 45). We suggest that in addition, the amassing of sphingolipids in NPC disease may also arise from the improper degradation and sensing of ORMDL proteins that in turn regulate de novo sphingolipid biosynthesis. Our results are consistent with a previous report that free cholesterol loading in macrophages induces translocation of ORMDL proteins from the ER to autophagosomes (28) and support the notion that ORMDLs are short-lived proteins that are degraded not only by the proteasome (43) but also by autophagic degradation (28). As accumulation of autophagosomes is a common feature among sphingolipidoses such as Gaucher disease (46), Fabry disease (47) and GM1 gangliosidosis (48), it is tempting to speculate that ORMDL mis-localization might also contribute to sphingolipid accumulation in those diseases.

Restoration of autophagic flux has been proposed to be a therapeutic avenue for treating NPC1 and other lysosomal disorders (31, 49, 50). Oral treatment of mice with NALL reduced p62 and LC3-II levels, restored autophagic flux, removed pathogenic protein aggregates, and mitigated neuronal cell death (18). Similarly, we found that treatment with NALL promotes the maturation of autophagosomes and the degradation of ORMDL cargo, preventing ORMDL accumulation in autophagosomes and likely restoring its ER localization. This correlated with the reduction of BMP accumulation and the build-up of complex sphingolipids and sphingosine, and importantly also decreased dihydrosphingosine and de novo sphingolipid biosynthesis. NALL also reduced perinuclear accumulation of lysosomes and lysosomal enlargement in NPC1-KO cells in line with a previous observation (34).

To date, there is no cure for NPC1 disease with only a few treatment options. Direct delivery of cyclodextrins to the CNS (51) and treatment with glucosylceramide synthase inhibitor Miglustat (52), have offered some promise despite several side effects. Recently, a promising treatment with NALL has been shown to improve neurological deficits and slow NPC1 disease progression (34, 35). In phase III clinical trials (NCT03759639), NALL was shown to be safe, well tolerated, and improved symptoms, functioning, and quality of life for pediatric and adult NPC1 patients (34, 35, 36, 53), and was recently submitted to the Food and Drug Administration and the European Medicines Agency for approval.

An orally bioavailable drug containing a racemic mixture of acetyl-leucine has been used for over 50 years in France for the treatment of vertigo (54). However, more recently the pharmacology of the drug has been attributed to the l enantiomer (55). Acetylation of leucine enables it to be more efficiently taken up into cells by organic anion transporters (OAT1 and OAT3) and monocarboxylate transporter type 1 (MCT1), instead of the L-type amino acid transporter (LAT1), explaining why NALL acts as a drug and its parent l-leucine does not (56). While the precise mechanism of NALL action in NPC1 disease is not yet clear, it was suggested that NALL may restore neuronal function and reduce neuroinflammation by altering glucose and antioxidant metabolism (34). Our results support the notion that restoration of autophagy flux mediated by NALL (18) and decreased accumulation of sphingolipids may also account for the beneficial effects observed in NPC1 patients and highlight the need for further understanding of its action and therapeutic potential.

## Data Availability

All data supporting this study are included in the manuscript and supplemental data.

## Supplemental data

This article contains supplemental data.

## Conflict of interest

The authors declare that they have no known competing financial interests or personal relationships that could have appeared to influence the work reported in this paper.
