# Supplementary material for: ORMDL mislocalization by impaired autophagy in Niemann-Pick type C disease leads to increased de novo sphingolipid biosynthesis
Source: J Lipid Res. 2024 May 6;65(6):100556. doi: 10.1016/j.jlr.2024.100556 (PMC11170278; doi:10.1016/j.jlr.2024.100556)
Supplement: Supplemental Data [file mmc1.pdf]

ORMDL MISLOCALIZATION BY IMPAIRED AUTOPHAGY IN NIEMANN-PICK TYPE C DISEASE LEADS TO INCREASED *DE NOVO* SPHINGOLIPID BIOSYNTHESIS

Ryan D. R. Brown[1], Usha Mahawar[1], Binks W. Wattenberg[1], and Sarah Spiegel[1*]

[1]Department of Biochemistry and Molecular Biology, Virginia Commonwealth University School of Medicine, Richmond, VA

SUPPLEMENTARY FIGURES

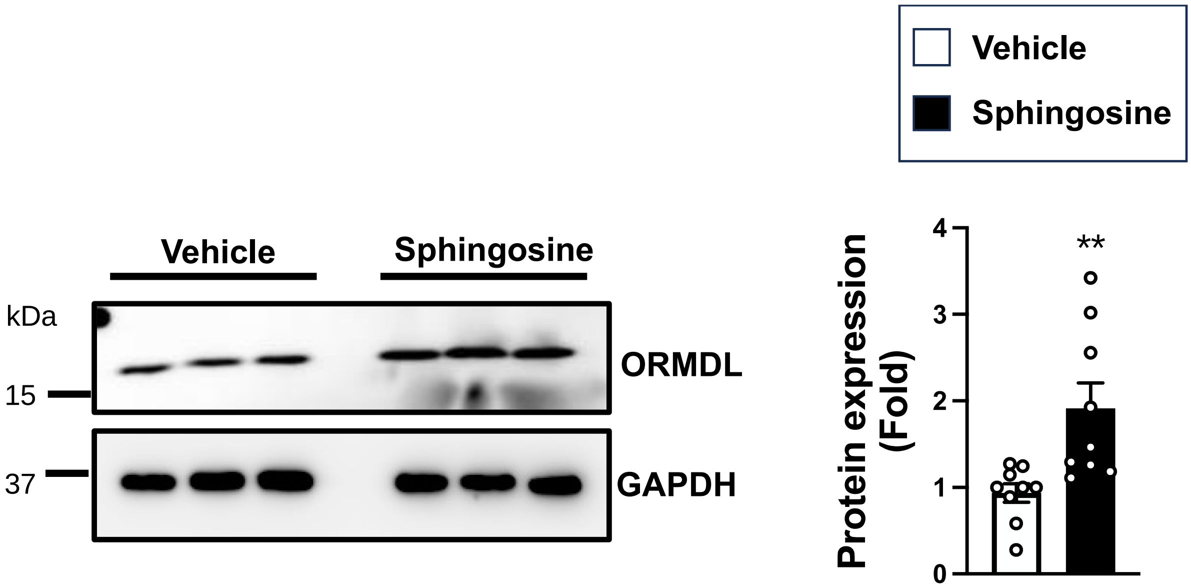

Supplementary Fig. 1. Sphingosine treatment increases ORMDL levels. WT HeLa cells were treated with vehicle or sphingosine (200 nM) for 24 hours and protein levels of ORMDLs were determined by immunoblotting. GAPDH was used as a loading control. Blots were quantitated by densitometry (N=3, n=3). Data are mean ± SEM. **p≤0.01 compared to vehicle. Unpaired two-tailed Student's t-test.

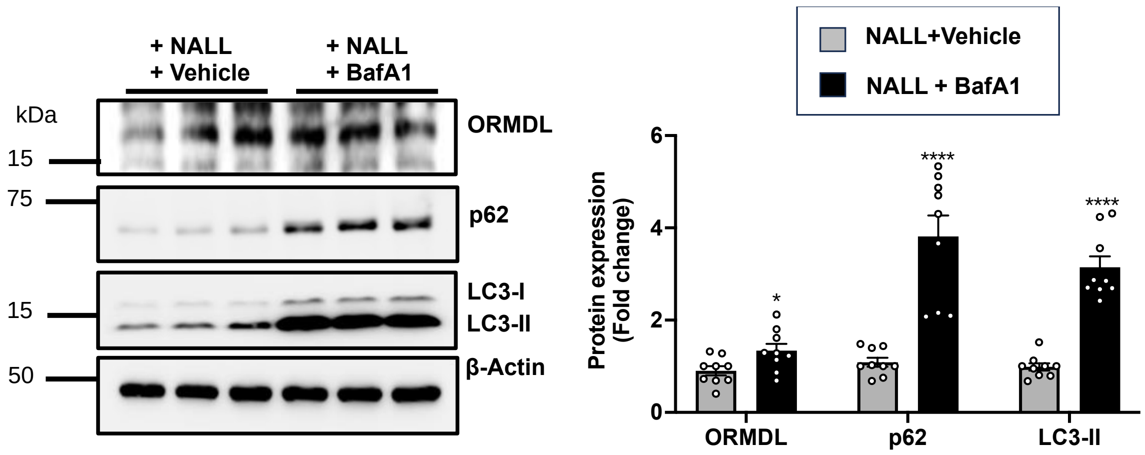

Supplementary Fig. 2. Bafilomycin A1 reversed the effect of NALL in NPC1 deleted cells. NPC1-KO cells were pretreated without or with BafA1 (0.5 µM) for 8 hours and then treated with NALL (5 µM) for 24 hours. Protein levels of ORMDL, p62 and LC3-II were determined by immunoblotting. β-actin was used as a loading control. Blots were quantitated by densitometry (N=3, n=3). Data are mean ± SEM. *p≤0.05 and ****p≤0.0001 compared to vehicle. Unpaired two-tailed Student's t-test.
